# Synthesis and Detailed Examination of Spectral Properties of (*S*)- and (*R*)-Higenamine 4′-*O*-β-d-Glucoside and HPLC Analytical Conditions to Distinguish the Diastereomers

**DOI:** 10.3390/molecules22091450

**Published:** 2017-08-31

**Authors:** Eisuke Kato, Ryohei Iwata, Jun Kawabata

**Affiliations:** Laboratory of Food Biochemistry, Division of Applied Bioscience, Graduate School of Agriculture, Hokkaido University, Kita-ku, Sapporo, Hokkaido 060-8589, Japan; ryochanboys@eis.hokudai.ac.jp (R.I.); junk@chem.agr.hokudai.ac.jp (J.K.)

**Keywords:** higenamine, tetrahydroisoquinoline, β-adrenergic receptor agonist, diastereomer separation

## Abstract

Higenamine is a tetrahydroisoquinoline present in several plants that has β-adrenergic receptor agonist activity. Study of the biosynthesis of higenamine has shown the participation of norcoclaurine synthase, which controls the stereochemistry to construct the (*S*)-isomer. However, when isolated from nature, higenamine is found as the racemate, or even the (*R*)-isomer. We recently reported the isolation of higenamine 4′-*O*-β-d-glucoside. Herein, its (*R*)- and (*S*)-isomers were synthesized and compared to precisely determine the stereochemistry of the isolate. Owing to their similar spectral properties, determination of the stereochemistry based on NMR data was considered inappropriate. Therefore, a high-performance liquid chromatography method was established to separate the isomers, and natural higenamine 4′-*O*-β-d-glucoside was determined to be a mixture of isomers.

## 1. Introduction

Higenamine (synonym norcoclaurine) is a tetrahydroisoquinoline found in several plants, including aconite root [1], lotus (*Nelumbo nucifera*) [2], *Gnetum parvifolium* [3], *Tinospora crispa* [4], and *Tinospora cordifolia* [5]. It possesses β-adrenergic receptor agonist activity owing to its structural similarity to catecholamine, has been shown to have a tracheal relaxation effect on guinea pig trachea [6]; it can protect against myocyte apoptosis in ischemia/reperfusion injury [7], collagen-induced arthritis, and spinal cord injury [8,9]; and it is clinically studied for therapeutic effects [10,11].

Biologically, higenamine is synthesized from dopamine and 4-hydroxyphenylacetic acid by the catalytic action of norcoclaurine synthase (NCS) [12,13]. The stereochemistry of higenamine is regulated by NCS, with its (*S*)-isomer known as the enzymatic product [12,13]. However, higenamine is often isolated from the plant as the racemate [3,14]. Furthermore, in the case of lotus, the (*S*)-enantiomer was isolated from the leaves [15], while the (*R*)-enantiomer was found in the seed embryo located near the plumule [16].

We previously reported the isolation of higenamine 4′-*O*-β-d-glucoside (**1**) from lotus plumule as a glucose uptake inducer against muscle cells, and indicated that its stereochemistry is (*S*) [17]. However, during a following research related to the structure-activity relationship study of **1**, a question arose about the stereochemistry of natural **1** isolated from lotus plumule, and the above reports have inspired us to precisely examine the stereochemistry of natural **1**.

As the correct assignment of stereochemistry in natural products is important, in this article, we chemically synthesized diastereomers **1*R*** and **1*S*** and compared their spectral properties. Careful examination of the spectral properties of the synthetic diastereomers indicated that distinguishing them using spectral methods was difficult. Therefore, we established high-performance liquid chromatography (HPLC) analysis conditions to separate the diastereomers, and used it to analyze the stereochemistry of natural **1** isolated from lotus plumule.

## 2. Results and Discussion

**Scheme 1:**
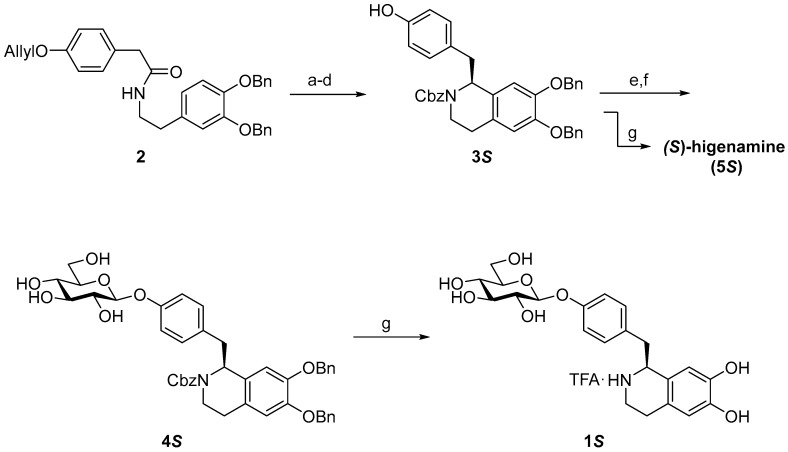
Synthesis of **1*S***. (**a**) POCl_3_, CHCl_3_, reflux; (**b**) RuCl[*R*,*R*-TsDPEN(*p*-cymene)], HCOOH, TEA, DMF; (**c**) CbzCl, TEA, DMAP, CH_2_Cl_2_; (**d**) Pd(Ph_3_P)_4_, K_2_CO_3_, MeOH, THF; (**e**) 2,3,4,6-tetra-*O*-acetyl-β-d-glucopyranosyl 2,2,2-trichloroacetimidate, BF_3_·Et_2_O, molecular sieves (4 Å), CH_2_Cl_2_; (**f**) K_2_CO_3_, MeOH, THF; (**g**) Pd(OH)_2_, H_2_, THF, MeOH.

To distinguish the diastereomers of **1** and determine their stereochemistry unequivocally, samples of **1*R*** and **1*S*** were required. Therefore, **1*R*** was selectively synthesized using a previously reported procedure and purified using reverse-phase HPLC [18], while **1*S*** was prepared using the same procedure by employing RuCl[*R*,*R*-TsDPEN(*p*-cymene)] as the catalyst for enantioselective reduction (Scheme 1).

Nuclear magnetic resonance (NMR) spectra of synthetic diastereomers **1*R*** and **1*S*** were obtained. However, in deuterium oxide, no apparent difference was found between the diastereomers (Table 1). This result might be due to (i) the stereochemistry of the synthetic products not being constructed as intended, resulting in the same diastereomer being isolated from both procedures; or (ii) the two diastereomers being indistinguishable under these NMR conditions.

**Figure 1:**
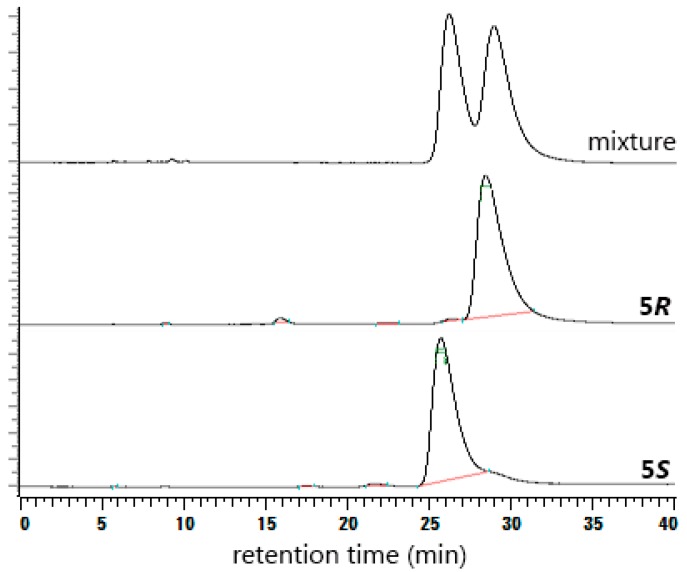
HPLC analysis of **5*R***, **5*S***, and their mixture. Conditions: column, Chiral CD-Ph (4.6 × 250 mm); mobile phase, 20 mM aq. KH_2_PO_4_/methanol = 50/50; flow rate, 0.5 mL/min; temperature, 40 °C; detection, 280 nm.

To avoid the former possibility, the stereochemistry of the higenamine moiety was confirmed by deprotection of **3*S*** and **3*R*** to obtain **5*R*** and **5*S***, respectively, followed by HPLC purification and specific rotation measurements. The specific rotation values (**5*S***: −20.0 (*c* = 0.68, methanol); **5*R***: +21.4 (*c* = 0.58, methanol)) were in good correlation with previous reports, which confirmed their stereochemistry [19,20]. Furthermore, the isomers were analyzed using a Chiral CD-Ph column (Shiseido Co., Tokyo, Japan) to determine their retention times (Figure 1). The specific rotations of synthetic **1*S*** (−32.4 (*c* = 1.5, methanol)) and **1*R*** (−16.3 (*c* = 1.5, methanol)) showed distinct values, which excluded the possibility of isomerization during the synthesis of **5*R*** and **5*S***.

**Figure 2:**
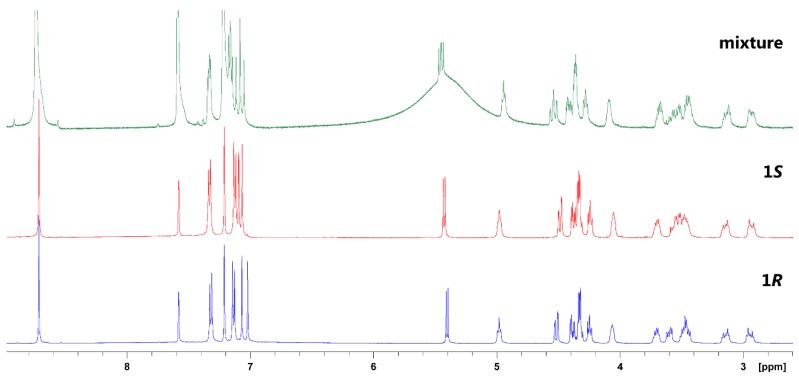
^1^H-NMR spectra of **1*R***, **1*S***, and their mixture (500 MHz, pyridine-*d*_5_, r.t.).

Deuterated solvents other than deuterium oxide were then tested for their suitability to separate the NMR signals of diastereomers of **1**. Methanol-*d*_4_ gave a result similar to that of deuterium oxide, and was therefore considered unsuitable (Table 2). In contrast, pyridine-*d*_5_ gave relatively separated ^1^H-NMR signals for the two diastereomers (Table 3). To determine whether these ^1^H-NMR signal differences in pyridine-*d*_5_ provided adequate evidence to determine the stereochemistry of **1**, a mixture of **1*R*** and **1*S*** was analyzed by ^1^H-NMR. The mixture showed distinguishable signals (Figure 2), but a problem was observed that prevented the use of NMR for the determination of stereochemistry.

As shown in Figure 2, when the ^1^H-NMR signals of the mixture were compared with those of **1*R*** or **1*S***, differences in the chemical shifts (ppm) were apparent. For example, the signal of the anomeric proton in glucose moiety (Glc-1) is 5.45 ppm for **1*R*** and 5.43 ppm for **1*S***; however, for the mixture of the two, the same proton appeared at 5.45 and 5.48 ppm, for which correspondence is unclear. These shifts in ppm value were presumably due to differences in the solution conditions, pH, or sample concentration during NMR analysis, which would influence the status of the cyclic amine in the tetrahydroisoquinoline moiety. They thus indicated that determining the stereochemistry of **1** through NMR experiments could potentially cause assignment errors, rendering it an inappropriate method.

Because of the fluctuation of ^1^H-NMR data with analytical conditions, distinguishing the diastereomers of **1** required an alternative method. HPLC is a method capable of separating and analyzing diastereomers. In HPLC analysis, analytical conditions are adjusted by changing the eluent used, which is present in large excess, and should therefore be a reliable method for analyzing the stereochemistry of **1**.

**Figure 3:**
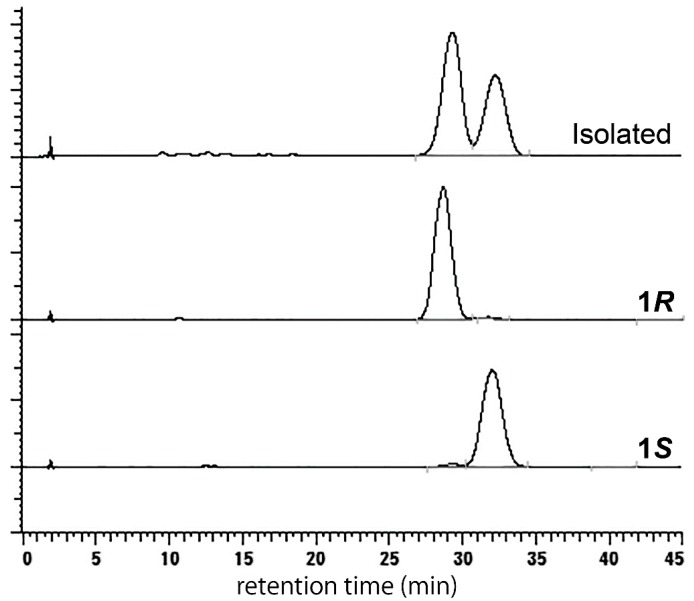
HPLC separation of diastereomers of **1**. Conditions: column, COSMOSIL Cholester (4.6 × 150 mm); mobile phase, 5% aq. methanol containing 0.1% trifluoroacetic acid; flow rate, 0.5 mL/min; detection, 280 nm.

Although a frequently employed C18 column was not capable of separating the isomers, a COSMOSIL Cholester column (Nakalai Tesque, Inc., Kyoto, Japan) was found to separate **1*R*** and **1*S*** to a good degree. Under the HPLC condition employed using this column, **1*R*** and **1*S*** showed apparent separation with retention times of 29 min and 32 min, respectively, indicating its ability to efficiently distinguish these isomers (Figure 3).

Natural **1**, isolated from lotus plumule, underwent stereochemical analysis using the established HPLC method. The results showed that natural **1** was a mixture of both isomers (**1*R***/**1*S*** = 59/41, Figure 3). This was further confirmed by analyzing the lotus plumule extract to avoid the possibility of isomerization during isolation, which showed a similar ratio (**1*R***/**1*S*** = 60/40, data not shown).

Other than lotus plumule, *Phoebe chekiangensis* has been reported to contain higenamine 4′-*O*-β-d-glucoside (**1**) [21]. The stereochemistry of **1** in *P. chekiangensis* was determined as (*S*) by Wu et al. by comparing with the NMR spectrum and specific rotation of synthetic **1*R*** [18]. Our results indicate that this result may need to be reassessed.

In conclusion, we synthesized **1*R*** and **1*S*** and directly compared their spectral data. We showed that the NMR spectra of these diastereomers were similar and their chemical shifts were influenced by solution conditions. This indicated that NMR was an unreliable method for determining the stereochemistry of **1**, and presumably those of other similar compounds. Therefore, an HPLC method for separating the diastereomers was established and natural **1**, isolated from lotus plumule, was analyzed and shown to be a mixture of diastereomers.

## 3. Materials and Methods

### 3.1. General

Chemicals were purchased from Wako Pure Chemical Industries, Ltd. (Osaka, Japan) unless otherwise noted. NMR spectra were obtained using a Bruker AMX 500 (Bruker BioSpin K.K., Yokohama, Japan) spectrometer, and residual solvents or *tert*-butanol were used as the internal standard (*tert*-butanol (deuterium oxide): ^1^H 1.24 ppm, ^13^C 30.29 ppm; methanol-*d*_4_: ^1^H 3.30 ppm, ^13^C 49.0 ppm; pyridine-*d*_5_: ^1^H 8.74 ppm, ^13^C 150.35 ppm). Mass spectra were obtained using a Waters LCT Premier Spectrometer (Waters Co., Milford, MA, USA). Specific rotations were measured using a Jasco P-2200 polarimeter (Jasco Co., Tokyo, Japan).

### 3.2. Synthesis

As the higenamine moiety with a protective group on the cyclic amine contains isomers, which were either rotamers resulting from the 4-hydroxybenzyl group or diastereomers resulting from the stereochemistry of the protective group on the cyclic amine, detailed data were not obtained for compounds **3** and **4**.

#### 3.2.1. (*S*)-*N*-Cbz-6,7-di-*O*-benzylhigenamine (**3*S***)

Compound **2** (1.03 g, 2.03 mmol), prepared using a reported procedure [18,22], was dissolved in chloroform (20 mL) and phosphoryl chloride (1.0 mL, 11.0 mmol) was added. The mixture was stirred for 17 h under reflux and then cooled to room temperature (r.t.), diluted with saturated NaHCO_3_ solution, and extracted with chloroform. The organic layer was washed with brine, dried over sodium sulfate, and concentrated. The residue was dissolved in dry *N*,*N*-dimethylformamide (DMF, 40 mL), RuCl[*R*,*R*-TsDPEN(*p*-cymene)] (35.5 mg, 0.0558 mmol, Kanto Co., Tokyo, Japan) was added, and the mixture was stirred under nitrogen. The solution was cooled to 0 °C, an azeotropic mixture of formic acid/triethylamine (TEA) (5:2 mole ratio, 1.6 mL) was added, and the mixture was stirred for 20 h at r.t. The reaction mixture was diluted with saturated NaHCO_3_ solution and extracted with ethyl acetate. The organic layer was washed with brine, dried over sodium sulfate, and concentrated. The resultant residue was dissolved in dichloromethane (20 mL) and triethylamine (1.0 mL, 7.2 mmol), and a catalytic amount of *N*,*N*-dimethyl-4-aminopyridine (DMAP) was added. Carbobenzoxy chloride (CbzCl, 0.3 mL, 2.1 mmol) was then added and the mixture was stirred for 1 h. The reaction mixture was dried, dissolved in ethyl acetate, and washed with 1 M aq. HCl and brine. The organic layer was dried over sodium sulfate and concentrated. The residue was dissolved in methanol (20 mL) and tetrahydrofuran (THF, 5 mL), following which Pd(PPh_3_)_4_ (59.1 mg, 0.051 mmol) and potassium carbonate (422.0 mg, 3.05 mmol) were added. The mixture was stirred for 2 h at 65 °C, cooled, and acidified with 1 M aq. HCl. The solution was extracted with ethyl acetate, washed with 1 M aq. HCl and brine, dried over sodium sulfate, and concentrated. The residue was purified with silica gel column chromatography (hexane/ethyl acetate = 2:1) to obtain **3*S*** (639.1 mg, 1.09 mmol, 54%). Electrospray ionization-mass spectrometry (ESI-MS) (positive): *m*/*z* 608 [M + Na]^+^, ${\left[\alpha \right]}_{D}^{27}$ −42.3 (*c* = 1.5, chloroform).

#### 3.2.2. (*S*)-*N*-Cbz-6,7-di-*O*-benzylhigenamine 4′-*O*-β-d-glucoside (**4*S***)

Compound **3*S*** (614.5 mg, 1.05 mmol) and 2,3,4,6-tetra-*O*-acetyl-β-d-glucopyranosyl 2,2,2-trichloroacetimidate (1.00 g, 2.34 mmol) were dissolved in dry dichloromethane (10 mL), powdered activated 4 Å molecular sieves (4 Å MS; 1.00 g) were added, and the mixture was stirred at −10 °C under nitrogen. BF_3_·Et_2_O (0.1 mL, 0.79 mmol) in dry dichloromethane (0.9 mL) was added and stirred for 30 min at −10 °C under nitrogen. The reaction mixture was diluted with saturated NaHCO_3_ solution and passed through a Celite pad. The organic layer was separated, washed with saturated NaHCO_3_ solution and brine, dried over sodium sulfate, and concentrated. The resultant residue was dissolved in methanol (10 mL) and THF (10 mL), then K_2_CO_3_ (301.8 mg, 2.18 mmol) was added, followed by stirring for 2 h. The reaction mixture was filtered, dried, and purified by silica gel column chromatography (chloroform/methanol = 9:1) to obtain **4*S*** (751.1 mg, 1.01 mmol, 96%). ESI-MS (positive): *m*/*z* 770 [M + Na]^+^, ${\left[\alpha \right]}_{D}^{26}$ −57.5 (*c* = 1.0, acetone).

#### 3.2.3. (*S*)-higenamine 4′-*O*-β-d-glucoside (**1*S***)

Compound **4*S*** (156.9 mg, 0.210 mmol) was dissolved in THF (10 mL) and methanol (2 mL), after which 20% palladium hydroxide on carbon (37.4 mg, Sigma-Aldrich Japan K.K., Tokyo, Japan) was added and stirred for 4 h under hydrogen. The reaction mixture was passed through a Celite pad, dried, and dissolved in 15% aq. methanol containing 0.1% trifluoroacetic acid (TFA). The solution was purified by HPLC (column, Inertsustain C18 (20 × 250 mm, GL Science Co., Tokyo, Japan); mobile phase, 15% aq. methanol containing 0.1% TFA; flow rate, 9.0 mL/min; detection, 280 nm) to obtain **1*S*** (107.2 mg, 0.196 mmol, 93%, 95% diastereomeric excess (*de*) from Figure 3) as a TFA salt. ESI-MS (positive): *m*/*z* 434 [M + H]^+^; ${\left[\alpha \right]}_{D}^{25}$ −32.4 (*c* = 1.5, methanol). See Table 1, Table 2 and Table 3 for NMR data.

#### 3.2.4. (*S*)-Higenamine (**5*S***)

Compound **3*S*** (24.6 mg, 0.042 mmol) was dissolved in THF (1 mL) and methanol (1 mL), 20% palladium hydroxide on carbon (5.4 mg, Aldrich Co.) was added, and the mixture was stirred for 16 h under hydrogen. The reaction mixture was passed through a Celite pad, dried, and dissolved in 30% aq. methanol containing 0.1% TFA (0.5 mL). The solution was purified by HPLC (column, Inertsustain C18 (20 × 250 mm, GL Science Co.); mobile phase, 30% aq. methanol containing 0.1% TFA; flow rate, 5.0 mL/min; detection, 280 nm) to obtain **5*S*** (13.2 mg, 0.034 mmol, 82%) as a TFA salt.

ESI-MS (positive): *m*/*z* 272 [M + H]^+^; ${\left[\alpha \right]}_{D}^{22}$ −20.0 (*c* = 0.68, methanol). ^1^H-NMR (500 MHz, methanol-*d*_4_, r.t.): 2.87–3.01 (3H, m), 3.21–3.27 (1H, m), 3.33–3.37 (1H, m), 3.42–3.47 (1H, m), 4.57 (1H, t, *J* = 5.0 Hz), 6.61 (1H, s), 6.62 (1H, s), 6.80 (2H, d, *J* = 10.0 Hz), 7.12 (2H, d, *J* = 10.0 Hz) ppm; ^13^C-NMR (125 MHz, methanol-*d*_4_, r.t.): 25.84, 40.62, 41.01, 58.06, 114.37, 116.33, 117.11, 123.79, 123.89, 127.14, 131.78, 145.92, 147.00, 158.35 ppm.

#### 3.2.5. (*R*)-Higenamine 4′-*O*-β-d-glucoside (**1*R***)

Compound **1*R*** was synthesized following the procedure detailed above for **3*S***, **4*S***, and **1*S***. Compound **3*S*** (yield 40% from **2**): ESI-MS (positive): *m*/*z* 608 [M + Na]^+^, ${\left[\alpha \right]}_{D}^{26}$ +44.9 (*c* = 1.5, chloroform); Compound **4*S*** (yield 56%): ESI-MS (positive): *m*/*z* 770 [M + Na]^+^, ${\left[\alpha \right]}_{D}^{26}$ +24.7 (*c* = 1.0, acetone); Compound **1*S*** (yield 87%, 95% *de* from Figure 3): ESI-MS (positive): *m*/*z* 434 [M + H]^+^; ${\left[\alpha \right]}_{D}^{25}$ −16.3 (*c* = 1.5, methanol). See Table 1, Table 2 and Table 3 for NMR data.

#### 3.2.6. (*R*)-Higenamine (**5*R***)

Compound **5*R*** was synthesized following the procedure detailed above for **5*S***. ESI-MS (positive): *m*/*z* 272 [M + H]^+^; ${\left[\alpha \right]}_{D}^{22}$ +21.4 (*c* = 0.58, methanol). The NMR spectrum was identical to that of **5*S***.

## Figures and Tables

**Table 1:** NMR data for synthetic **1*R*** and **1*S*** in deuterium oxide.

No.	Synthetic 1*R*		Synthetic 1*S*	
	δ_H_	δ_C_	δ_H_	δ_C_
1	4.64 (dd, *J* = 5.7, 8.8 Hz)	56.78	4.61 (dd, *J* = 5.7, 9.1 Hz)	56.75
3	3.46–3.52 (m)	39.93	3.47 (td, *J* = 6.3, 12.6 Hz)	39.90
	3.28 (td, *J* = 6.3, 13.2 Hz)		3.27 (td, *J* = 6.3, 12.6 Hz)	
4	2.96–3.00 (m)	24.69	2.95–3.00 (m)	24.67
	2.91 (td, *J* = 6.3, 17.3 Hz)		2.90 (td, *J* = 6.3, 17.3 Hz)	
5	6.74 (s)	116.49	6.73 (s)	116.48
6 or 7	-	143.53	-	143.51
6 or 7	-	144.77	-	144.76
8	6.66 (s)	114.53	6.64 (s)	114.50
9 or 10	-	123.84	-	123.79
9 or 10	-	124.47	-	124.45
1′	-	130.30	-	130.27
2′, 6′	7.26 (2H, d, *J* = 8.5 Hz)	131.61	7.25 (2H, d, *J* = 8.6 Hz)	131.58
3′, 5′	7.13 (2H, d, *J* = 8.5 Hz)	117.72	7.12 (2H, d, *J* = 8.6 Hz)	117.74
4′	-	156.74	-	156.75
7′	3.00 (dd, *J* = 8.8, 14.5 Hz)	39.13	2.97 (dd, *J* = 9.1, 14.5 Hz)	39.14
	3.40 (dd, *J* = 5.7, 14.5 Hz)		3.36 (dd, *J* = 5.7, 14.5 Hz)	
Glc-1	5.12 (d, *J* = 7.6 Hz)	100.78	5.09 (d, *J* = 7.3 Hz)	100.84
Glc-2	3.58 (dd, *J* = 7.6, 9.5 Hz)	73.59	3.58 (dd, *J* = 7.3, 9.5 Hz)	73.60
Glc-3	3.50 (dd, *J* = 8.6, 9.5 Hz)	76.25	3.50 (dd, *J* = 9.1, 9.5 Hz)	76.26
Glc-4	3.61 (dd, *J* = 8.6, 9.5 Hz)	70.11	3.61 (dd, *J* = 8.2, 9.1 Hz)	70.12
Glc-5	3.63 (ddd, *J* = 1.9, 5.7, 9.5 Hz)	76.82	3.60–3.63 (m)	76.81
Glc-6	3.93 (dd, *J* = 1.9, 12.5 Hz)	61.23	3.93 (dd, *J* = 2.2, 12.6 Hz)	61.26
	3.75 (dd, *J* = 5.7, 12.5 Hz)		3.76 (dd, *J* = 5.7, 12.6 Hz)	

**Table 2:** NMR data for synthetic **1*R*** and **1*S*** in methanol-*d*_4_.

No.	Synthetic 1*R*		Synthetic 1*S*	
	δ_H_	δ_C_	δ_H_	δ_C_
1	4.61 (dd, *J* = 6.1, 8.4 Hz)	57.78	4.59 (dd, *J* = 5.8, 8.2 Hz)	57.75
3	3.42–3.48 (m)	40.87	3.42–3.48 (m)	40.82
	3.25 (td, *J* = 6.2, 13.0 Hz)		3.25 (td, *J* = 6.4, 13.0 Hz)	
4	2.98 (td, *J* = 6.2, 17.3 Hz)	25.69	2.98 (td, *J* = 6.4, 17.3 Hz)	25.64
	2.90 (td, *J* = 6.2, 17.3 Hz)		2.90 (td, *J* = 6.4, 17.3 Hz)	
5	6.62 (s)	116.21	6.62 (s)	116.21
6 or 7		158.69		158.61
6 or 7		146.91		146.82
8	6.58 (s)	114.25	6.56 (s)	114.27
9 or 10		123.65		123.70
9 or 10		123.60		123.60
1′		130.29		130.32
2′, 6′	7.23 (2H, d, *J* = 8.6 Hz)	131.64	7.23 (2H, d, *J* = 8.6 Hz)	131.66
3′, 5′	7.11 (2H, d, *J* = 8.6 Hz)	118.43	7.11 (2H, d, *J* = 8.6 Hz)	118.37
4′		158.69		158.61
7′	3.35–3.42 (m)	40.43	3.35–3.42 (m)	40.40
	3.03 (dd, *J* = 8.4, 14.5 Hz)		3.04 (dd, *J* = 8.2, 14.4 Hz)	
Glc-1	4.90 (d, *J* = 7.6 Hz)	102.30	4.90 (d, *J* = 7.7 Hz)	102.27
Glc-2,3,5	3.42–3.48 (3H, m)	78.20	3.42–3.48 (3H, m)	78.12
		78.01		77.94
		74.87		74.85
Glc-4	3.35–3.42 (m)	71.43	3.35–3.42 (m)	71.38
Glc-6	3.89 (dd, *J* = 2.2, 12.0 Hz)	62.59	3.89 (dd, *J* = 2.3, 12.0 Hz)	62.53
	3.68 (dd, *J* = 5.8, 12.0 Hz)		3.68 (dd, *J* = 5.8, 12.0 Hz)	

**Table 3:** NMR data for synthetic **1*R*** and **1*S*** in pyridine-*d*_5_.

No.	Synthetic 1*R*		Synthetic 1*S*	
	δ_H_	δ_C_	δ_H_	δ_C_
1	5.00 (t, *J* = 5.7 Hz)	57.11	5.00 (t, *J* = 6.5 Hz)	57.05
3	3.73 (ddd, *J* = 6.0, 6.5, 12.3 Hz)	40.19	3.73 (ddd, *J* = 6.0, 6.3, 12.5 Hz)	40.00
	3.46–3.62 (m)		3.51 (ddd, *J* = 6.0, 6.3, 12.5 Hz)	
4	3.17 (ddd, *J* = 6.5, 6.5, 14.0 Hz)	25.91	3.17 (ddd, *J* = 6.3, 6.3, 17.0 Hz)	25.81
	2.96 (ddd, *J* = 6.0, 6.0, 14.0 Hz)		2.97 (ddd, *J* = 6.0, 6.0, 17.0 Hz)	
5	7.12 (s)	116.98	7.09 (s)	116.98
6	-	146.69	-	146.62
7	-	147.79	-	147.82
8	7.09 (s)	115.04	7.05 (s)	115.00
9	-	124.36	-	124.36
10	-	124.20	-	123.64
1′	-	130.61	-	130.62
2′, 6′	7.36 (2H, d, *J* = 8.4 Hz)	131.74	7.34 (2H, d, *J* = 8.5 Hz)	131.75
3′, 5′	7.15 (2H, d, *J* = 8.4 Hz)	117.56	7.16 (2H, d, *J* = 8.5 Hz)	117.53
4′	-	158.21	-	158.23
7′	3.46–3.62 (2H, m)	40.40	3.63 (dd, *J* = 6.5, 14.2 Hz)	40.48
	-		3.48 (dd, *J* = 6.5, 14.2 Hz)	
Glc-1	5.45 (d, *J* = 7.9 Hz)	102.44	5.43 (d, *J* = 7.7 Hz)	102.53
Glc-2	4.27 (dd, *J* = 7.9, 8.8 Hz)	75.27	4.27 (dd, *J* = 7.7, 9.1 Hz)	75.27
Glc-3, 4	4.33–4.37 (2H, m)	78.83	4.33–4.38 (2H, m)	78.86
	-	71.62	-	71.64
Glc-5	4.06–4.09 (m)	79.18	4.07–4.10 (m)	79.25
Glc-6	4.51 (dd, *J* = 2.2, 12.0 Hz)	62.71	4.54 (dd, *J* = 2.2, 12.0 Hz)	62.74
	4.40 (dd, *J* = 5.0, 12.0 Hz)		4.41 (dd, *J* = 5.0, 12.0 Hz)	
